# Two Doses to ICU or When Lactate Equals Glucose: Life‐Threatening Lactic Acidosis due to Metformin After Initiation of Semaglutide

**DOI:** 10.1155/crcc/3478800

**Published:** 2026-05-16

**Authors:** Zhanna Albany, David Roe, Daniel Gutteridge, Hanna Webb, Areeba Kara

**Affiliations:** ^1^ Department of Medicine, Indiana University School of Medicine, Indianapolis, Indiana, USA, indiana.edu

## Abstract

Glucagon‐like peptide 1 agonists have emerged as a promising treatment option for diabetes. As the prescription of these agents is rapidly increasing, clinicians will need to familiarize themselves with the potential adverse events associated with their use. Nausea, vomiting, and poor oral intake are known side effects of GLP‐1 agonists. In elderly patients with diabetes and multiple comorbidities who are on complex medication regimens, semaglutide can lead to clinically significant dehydration at a rapid pace. We report a case of a patient developing dehydration leading to acute kidney failure with life‐threatening metformin‐induced lactic acidosis. Close clinical and laboratory monitoring is advised when initiating semaglutide treatment in combination with other diabetic medications, especially in elderly patients.

**Learning Points**

• Close clinical and laboratory monitoring is advised when initiating semaglutide treatment in combination with other diabetic medications such as metformin, insulin, sulfonylureas, diuretics, ACE, or ARB inhibitors.
• Kussmaul breathing is an important early clinical indicator of severe metabolic acidosis and should prompt immediate laboratory evaluation.
• Elderly patients are likely at an increased risk of developing dehydration with semaglutide.
• Acute kidney failure due to dehydration secondary to semaglutide and concurrent use of metformin may lead to life‐threatening lactic acidosis.
• Proactive patients′ education regarding temporary discontinuation of metformin during acute illness or dehydration (sick day rule) is an important preventative strategy.

## 1. Background

Driven largely by diabetes mellitus (DM) Type 2, the prevalence of people living with DM worldwide is projected to double from 529 million in 2021 to 1.31 billion people by 2050. [1, 2]. Glucagon‐like peptide 1 (GLP‐1) agonists have emerged as a promising treatment option for DM that also lead to weight loss and have proven cardiovascular benefit [1]. As the prescription of these agents is rapidly increasing, clinicians will need to familiarize themselves with the potential adverse events associated with their use. The number of patients on semaglutide showed a 40‐fold increase between 2019 and 2022 in a large statewide health care system demonstrating the exponential growth in GLP‐1 prescribing [2]. Semaglutide is the only GLP‐1 agonist currently available as a subcutaneous and oral formulation. Randomized controlled trials (RCTs) have demonstrated an overall favorable safety profile with low risks of severe adverse effects such as acute kidney injury (AKI) [3]. Potent glucose‐lowering effects may lead to hypoglycemia especially when semaglutide is used in combination with insulin. If patients are simultaneously treated with metformin in addition to insulin and semaglutide, the resulting nausea and poor oral intake can precipitate dehydration, hypoglycemia and AKI with serious clinical implications.

## 2. Case presentation

A 77‐year‐old man with Type 2 diabetes for more than 30 years presented to the emergency department (ED) with nausea, vomiting and hypoglycemia. He had been adherent to a carbohydrate‐controlled diet, long‐acting insulin glargine 12 units subcutaneously twice a day, glimepiride 4 mg and metformin 1000 mg orally twice a day for many years without complications. His most recent hemoglobin A1c level was 6.9%. As the patient was interested in weight loss, glimepiride was stopped and low dose semaglutide 0.25 mg weekly subcutaneously was initiated 3 weeks prior to ED presentation. His medications also included lisinopril‐hydrochlorothiazide 20–12.5 mg daily, atorvastatin 20 mg daily, amitriptyline 25 mg at bedtime and terazosin 5 mg at bedtime for hypertension, dyslipidemia, insomnia, and benign prostatic hyperplasia, respectively. He was not taking aspirin, NSAIDs, salicylates, or any herbal medications. He experienced nausea following the first dose of semaglutide, with episodes of confusion corresponding to blood glucose (BG) below 70 at home. He reported these hypoglycemic episodes to his endocrinologist and insulin was discontinued. After the second dose of semaglutide, his nausea, poor oral intake, and vomiting worsened, and he developed constipation. He was brought in via ambulance with the chief complaint of altered mental status after he experienced a near syncope and his BG measured at home was 30. In the ED his vitals were: temperature 34.8°C (rectal), blood pressure 120/55, heart rate 108, respirations 36, oxygen saturation 96% breathing room air. Physical examination revealed dry mucous membranes, deep and rapid breathing consistent with Kussmaul breathing pattern, and impaired cognition with slow responses. The remainder of the physical exam was unrevealing.

### 2.1. Investigations

Laboratory tests were remarkable for sodium 139 mmol/L, potassium 4.3 mmol/L, chloride 99 mmol/L, CO2 7 mmol/L, anion gap 33 mmol/L, blood urea nitrogen 33 mg/dL, creatinine 1.38 mg/dL (2 weeks prior to presentation creatinine was 0.9 mg/dL), lactic acid 18 mmol/L, glucose 18 mg/dL, serum osmolarity 308 mOsm/kg, with normal liver enzymes, normal thyroid stimulating hormone level and arterial blood gas pH 7.02, PCO2 11 mmHg, PO2 78 mmHg. His weight had decreased 4 kg in the 1 month preceding this presentation. His creatinine increased from 0.9 1 month prior to admission to 1.43 on presentation indicating Stage 1 AKI according to KDIGO criteria [4]. His urinalysis was notable for 100 ketones and 3‐5 hyaline casts. His urine electrolytes were sent after he had received bicarbonate fluids, therefore limiting their diagnostic utility for prerenal state. Transthoracic echocardiogram demonstrated normal left ventricular systolic function with no wall motion abnormalities. CT abdomen and pelvis showed a large burden of stool in the colon and otherwise no acute findings.

### 2.2. Differential Diagnosis

Type A lactic acidosis, which is more common, develops as a consequence of tissue hypoxia due to any cause (e.g., sepsis, cardiac failure or arrest, respiratory failure, hypoperfusion due to hypovolemia). Type B lactic acidosis occurs when lactate accumulates without evidence of tissue hypoperfusion such as when there is either increased production or impaired clearance of lactate related to liver failure, or exposure to certain medications or toxins. Metformin‐associated lactic acidosis is categorized as Type B lactic acidosis, which does not involve tissue hypoxia. However, the two types frequently overlap in clinical practice. Metformin‐induced lactic acidosis has been attributed to several factors. Metformin inhibits mitochondrial function leading to increased lactate production via anaerobic glycolysis. The liver clears lactate through gluconeogenesis, which metformin impairs thus increasing lactate accumulation. Since metformin and lactate are primarily excreted by the kidneys [5] any concomitant renal impairment leads to increased levels resulting in worsening acidosis. Both the initial level of lactate and the rate of decrease are strong predictors of survival [6]. Additional risk factors for lactic acid accumulation when treated with metformin include active alcohol use, concurrent liver disease, acute heart failure and hypoxic states. Our patient had no reported alcohol use, liver function tests were normal, and he had no history of heart failure. Echocardiogram showed normal cardiac function, ruling out cardiogenic causes of lactic acidosis. He had no evidence of infection, sepsis, hypothyroidism or hypotension. Therefore, it is likely that nausea, vomiting and poor oral intake triggered by semaglutide contributed to dehydration resulting in AKI that led to metformin toxicity with resulting life‐threatening lactic acidosis.

### 2.3. Treatment

The patient was started on bicarbonate infusion and admitted to the intensive care unit where emergent hemodialysis was initiated within hours of admission for severe refractory acidemia with a pH less than 7.1 and lactate above 16. A dialysate bicarbonate level of 35 was used, with a blood flow rate of 350 mL/minute and a dialysate flow rate of 700 mL/minute. His lactate and serum pH levels were monitored throughout the treatment, and dialysis was discontinued after 210 min. At that time, his lactate was 7 and serum pH was 7.4. He did not require any further hemodialysis treatments. He maintained his urine output and after 2 days the lactate level improved to 3.4. Semaglutide, metformin, and lisinopril‐hydrochlorothiazide were discontinued on admission. His nausea and vomiting resolved, and oral intake normalized.

### 2.4. Outcome and Follow‐Up

Our patient had a remarkable recovery. At the time of discharge, he was off hemodialysis and his creatinine was 0.8 mg/dL. He was discharged on low dose long‐acting insulin. At a 3‐week follow‐up visit, his renal function and lactate remained normal, creatinine 0.84 mg/dL, and lactate 1.6 mmol/L.

## 3. Discussion

Although metformin has been in clinical use for more than half a century, semaglutide is one of the more recently approved agents in the glucagon‐like peptide receptor agonist (GLP‐1 agonist) class. Overall, semaglutide has been shown to have a low risk of severe adverse events in line with other GLP‐1 agonists. Adverse events associated with semaglutide include hypoglycemia, gastrointestinal side effects including nausea, vomiting, diarrhea, AKI, pancreatitis, gallbladder events, medullary thyroid carcinoma, and diabetic retinopathy progression [3].

Nausea and vomiting are the most common gastrointestinal side effects experienced by patients treated with GLP‐1 agonists with up to a quarter of patients reporting these symptoms. [3]. It is unclear if metformin addition to semaglutide makes nausea and vomiting worse versus semaglutide alone. A meta‐analysis examining 32 RCTs demonstrated that the background use of metformin was associated with more nausea and vomiting when combined with GLP‐1 receptor agonists [7]. Conversely, rates of nausea and vomiting reported with semaglutide in combination with metformin in SUSTAIN‐2 trials were slightly lower at 18%–20% and 6–8%, respectively, compared with nausea (20%–24%) and vomiting (10%–13%) occurrence with semaglutide alone in SUSTAIN‐1 clinical trial [8, 9]. In a pooled meta‐analysis of 10 RCTs examining overweight or obese patients with DM, no significant differences in the occurrence of gastrointestinal symptoms were found between those using semaglutide combined with metformin versus those using metformin alone [10].

Semaglutide induced anorexia, nausea and vomiting can lead to dehydration, especially among older patients who are sensitive to fluid balance alterations. Direct rates of clinically significant dehydration have not been reported. Using AKI as an indirect indicator of volume status, AKI occurrence was reported to be similar between the semaglutide and placebo groups [8]. The mean ages of the patients in the SUSTAIN 1 and 2 trial receiving semaglutide were 54 and 55 years, respectively. As the use of these agents increases, including in patients such as ours who are older and with higher burdens of comorbidities, the rates of adverse events may also increase. In our patient, semaglutide initiation along with ongoing treatment with metformin, insulin, glimepiride, lisinopril and hydrochlorothiazide highlights the important role of polypharmacy that contributed to and propelled AKI. Similar to our case, acute renal failure has been reported when exenatide was coadministered with diuretics and angiotensin II blockers [11].

The presence of Kussmaul breathing observed in our patient serves as an important early clinical indicator of severe metabolic acidosis, appearing before complete biochemical analysis is available. It highlights the importance of recognizing compensatory respiratory patterns in patients presenting with altered mental status. Severe hypoglycemia likely contributed to altered mentation and was confounded by recent use of insulin and sulfonylurea. The urine ketones detected we believe were secondary to starvation ketosis rather than diabetic ketoacidosis, given the profound hypoglycemia, absence of insulin therapy, and the primary lactic acidosis mechanism.

We used the Naranjo scale (see Table 1) to assess the link between dehydration and semaglutide use, scoring a total of 3 points indicating a possible drug‐related adverse reaction. Case reports are frequently the first signals of potential safety risks post marketing. To our knowledge, there are limited prior reports of this specific complication. Halderman et al. described a case of AKI induced by oral semaglutide leading to metformin‐associated lactic acidosis [12]. That case, along with the present report, suggests this may be an underrecognized clinical syndrome as semaglutide prescribing expands to older patients with more complex medication regimens. Based on our patient′s clinical presentation we suspect that he developed dehydration after semaglutide initiation, exacerbated by the second dose and his antihypertensive regimen. The development of AKI with the continued use of metformin, lead to severe life‐threatening lactic acidosis that contributed to his kidney injury thus perpetuating the vicious cycle.

**Table 1:** Naranjo scale: Dehydration due to nausea and poor oral intake after semaglutide initiation.

Naranjo Adverse Drug Reaction Probability Scale				
Question/answer	Yes	No	Do not know	Score
Are there previous conclusive reports on this reaction? *No*	+1	0	0	0
Did the adverse event appear after the suspected drug was administered? *Yes*	+2	−1	0	2
Did the adverse reaction improve when the drug was discontinued, or a specific antagonist was administered? *Yes*	+1	0	0	1
Did the adverse effect re‐appear when the drug was re‐administered? *Semaglutide was not re-administered*	+1	0	0	0
Are there alternative causes (other than the drug) that could on their own have caused the reaction? *Yes several medications such as lisinopril, hydrochlorothiazide, metformin were used that could have contributed or compounded dehydration*	−1	+2	0	−1
Did the reaction reappear when a placebo was given? *Placebo was not given*	−1	+1	0	0
Was the drug detected in blood or other fluids in concentrations known to be toxic? *Semaglutide level was not measured*	+1	0	0	0
Was the reaction more severe when the dose was increased or less severe when the dose was decreased? *The dose was not changed*	+1	0	0	0
Did the patient have a similar reaction to the same or similar drugs in any previous exposure? *Not applicable*	+1	0	0	0
Was the adverse effect confirmed by any objective evidence? *Yes dry mucous membranes and osmolarity measured*	+1	0	0	1
Total score:				3

When initiating semaglutide in setting of advanced age and complex medication regimen, close monitoring for AKI and metabolic derangements is recommended. Normal range GFR in older patients with long‐standing DM and chronic kidney disease should not be seen as reassuring and does not eliminate the need for close laboratory monitoring. Elderly patients are likely to have an elevated risk of developing dehydration with semaglutide.

## Author Contributions

D.R., H.W., and Z.A. were involved in patient care and manuscript conceptualization. Z.A. and A.K. wrote the manuscript. D.G., D.R., H.W., and A.K. reviewed and edited the manuscript.

## Funding

No funding was received for this manuscript.

## Disclosure

All authors approved the final version.

## Consent

Patient consent was sought and provided prior to writing. All institutional guidance on best practices was followed in the writing of this case report.

## Conflicts of Interest

The authors declare no conflicts of interest.

## Data Availability

The data that supports the findings of this study is available in the supplementary material of this article.
